# On the resuscitation of clinical freedom

**DOI:** 10.1186/1472-6963-10-184

**Published:** 2010-06-28

**Authors:** Amanda Burls

**Affiliations:** 1 Department of Primary Health Care, University of Oxford, Old Road Campus, Oxford OX3 7LF, UK

## Abstract

**Background:**

This paper is a response to the suggestion by Sacristán *et al *that clinicians can increase their clinical freedom by undertaking individualised economic analyses that demonstrate that interventions, which at a population level do not reach conventional thresholds of cost-effectiveness, do so in particular patients.

**Discussion:**

In this reply, I question the presumption that "clinical freedom" is necessarily desirable and go on to argue that, even if it is, the proposal that clinicians should do individualised economic evaluation is flawed. Firstly, the additional clinical choice that may be gained from individualised economic analyses that demonstrate that an intervention, generally considered not to be cost-effective, is cost-effective in a particular patient, is likely to be counterbalanced by other analyses that produce the converse result (i.e. that an intervention that is cost-effective at a population level may not be so in a particular patient) - a complementary consequence, which is ignored by Sacristán *et al *in their paper. Secondly, the skills and time required to do an individualised economic analysis are likely to exceed those of most clinicians. Thirdly, and most importantly, asking clinicians to make rationing judgements at the point of care is a threat to patient trust and can harm the doctor-patient relationship.

**Summary:**

Individualised economic evaluations are neither a desirable nor feasible method for increasing clinical choice.

## Background

This paper is a response to the suggestion made by Sacristán *et al *in this publication that clinicians can increase their clinical freedom by undertaking individualised economic analyses to demonstrate that interventions, which at a population level do not reach conventional thresholds of cost-effectiveness, do so in particular patients [1].

## Discussion

### Clinical Freedom

When in 1983 Hampton declared that clinical freedom - *"the right... of doctors to do whatever in their opinion was best for their patients" - *was dead, he added:

*"Clinical freedom should, however, have been strangled long ago, for at best it was a cloak for ignorance and at worst an excuse for quackery. Clinical freedom was a myth that prevented true advance."*[2]

In the early days of evidence-based medicine (EBM), some opposed it on the grounds that it threatened clinical freedom. However, the absurd notion that knowledge, because it reduces the number of choices that can be sensibly made, somehow erodes freedom was soon laid to rest [3]. It was not a hard battle: the ridiculousness of equating ignorance with freedom was derided long before the phrase "evidence-based medicine" was coined. As Archie Cochrane wrote while reflecting on what he had learnt as prisoner of war:

*"What I decided I could not continue doing was making decisions about intervening (for example pneumothorax and thoracoplasty) when I had no idea whether I was doing more harm than good. I remember reading a pamphlet (I think from the BMA) extolling the advantages of the freedom of British doctors to do whatever they thought best for their patients. I found it ridiculous. I would willingly have sacrificed all my medical freedom for some hard evidence telling me when to do a pneumothorax ...*" [4]

### Individualised economic analyses

Nonetheless today Sacristán *et al *resurrect the idea that *"Evidence-based medicine ... has contributed to augment the feeling that clinicians play a secondary role in the therapeutic decision process*"[1] and propose a new approach to restore clinical freedom.

The approach proposed is the use of cost-effectiveness analysis at the individual patient level - a suggestion that will be counterintuitive, and perhaps even abhorrent, to many clinicians: most clinicians would not relish telling their patient that they have chosen not to give him or her an effective treatment because the resource required would benefit someone else more. Given that efficiency decisions are about saying "no" to some interventions in some circumstances, can using economic evaluations enhance clinical freedom as Sacristán *et al *suggest? While it may be easy to convince clinicians that ignorance is undesirable, and what we need are treatments that have been shown to be effective, it is harder to convince them that denying patients effective treatments, on the grounds that they are too expensive, enhances clinical care. As one paediatrician proudly proclaimed at a National Institute for Health and Clinical Excellence (NICE) meeting I attended, *"I am not interested in economics - I am a doctor"*. Even Archie Cochrane, when campaigning in the 1930s to get a National Health Service, chose the slogan: *"All effective treatments must be free" *for his banner [4].

Part of the reason why Sacristán *et al *think that individualised cost-effectiveness analyses can enhance clinical freedom may be because they almost totally ignore the fact that economic evaluations may imply that a patient should not be treated. Instead they focus solely on examples where a policy decision not to recommend a treatment because it does not reach an acceptable willingness-to-pay threshold could be challenged on the grounds that this is not the case for a particular patient. It is this special pleading, using economic arguments to establish that one's own patient is different, that Sacristán *et al *envisage will furnish clinicians with the vaunted clinical freedom.

Ever since NICE's inception, which formally added the "fourth hurdle" of having to demonstrate that an intervention is cost-effective before it is recommended for use in the NHS, when there has been a negative decision about a new intervention, sponsoring organisations have tended to argue either that the cost-effectiveness threshold is too low in this particular circumstance or condition or that the incremental cost-effectiveness ratio (ICER) of their product really lies within a range considered to represent a good use of health resources. (Industry sponsored economic evaluations tend to make overly optimistic assumptions resulting in ICERs that are much more favourable to their product than those produced by independent researchers [5]). The pharmaceutical industry has often battled against policy decisions not to recommend interventions that are not cost-effective and sought to reverse or get around them by arguing, for example, that there are sub-groups in which an intervention is indeed cost-effective, or that patient utilities are different from those that were used to inform the policy decision. Sacristán *et al *take a similar approach but suggest that clinicians apply these arguments at the individual patient level. They illustrate their case by using concrete examples in which an intervention becomes cost-effective in an individual patient because of their greater potential for benefit or likelihood of adherence, or because a different comparator (i.e. the alternative treatment that would have been used in the particular patient or context) is more appropriate, or because the patient has different values (e.g. utility of different outcomes).

Are Sacristán *et al *right that this approach could increase the treatment choices available to clinicians? Certainly they are right that some treatments are likely to be cost-effective in some people but not in others. Palivizumab, a prophylactic treatment to prevent serious illness from respiratory syncytial virus (RSV), is a good example. In most adults and children RSV infection is asymptomatic or produces mild respiratory illness, and palivizumab is expensive. In some children, however, RSV can lead to life-threatening illness and death. The key to the cost-effective use of palivizumab is being able to identify the subset of children who are likely to be at most risk of serious illness [6]. If we can do this, we can treat this group even though the drug is not a good use of money when used unselectively in the licensed indication. However the counterpart to recognising that it is a good use of resources in some, is the recognition that it is a poor use of resources in others. Sacristán *et al*'s arguments tend to be lopsided, however, focusing mainly on examples of why an intervention that is not generally cost-effective could reach an accepted willingness-to-pay threshold in some patients. They neglect the complementary and converse situations when a generally cost-effective intervention would not be cost-effective for a particular patient. Moreover, their proposal is unrealistic - most clinicians do not have the skills or time to undertake an individualised economic evaluation to establish the effect of different risk factors and utilities. The palivizumab economic evaluation [6] required skilled systematic reviewers, health information specialists, statisticians and mathematical modellers to identify and define the sub-groups in which the drug reaches conventional levels of cost-effectiveness. To attempt this at an individual clinician/patient level, if not unfeasibly demanding, would inevitably carry a large opportunity cost in time for a clinician.

I believe that Sacristán *et al *also fail to appreciate fully the nature of the doctor-patient relationship. Doctors need to be able to relate to the individual patients in front of them as their strong advocate, treating them in a way that, after duly taking into account the patient's values and the constraints of service within which they are working, will produce maximal benefit for the patient. Requiring doctors to make rationing decisions at the point of care has the potential to seriously undermine the doctor-patient relationship and patient trust [7]. Therefore, I would argue that rationing decisions are best made at a population level, where the opportunity costs can be appropriately taken into account. The analyses on which such policy decisions are based should include consideration and evaluation of variable risk factors and utilities that might enable identification of sub-groups in which the estimated ICERs differ to a degree that has different implications for practice. The policy decisions that result from this process create and define the constraints within which clinicians must then work and strive to do the very best for their patients. (This could include informing a patient that there are potentially effective treatments that cannot be prescribed within the service in which care is being provided.)

Given Hampton's observation that clinical freedom died *"crushed between the rising cost of new forms of investigation and treatment and the financial limits inevitable in an economy that cannot expand indefinitely"*[2], it is ironic that the very methods used to manage the tension are now being proposed as the means for its resuscitation.

## Summary

Individualised economic evaluations are neither a desirable nor feasible method for increasing clinical choice: additional clinical choices gained from individualised economic analyses are likely to be outweighed by those producing the converse result; the skills and time required are likely to exceed those of most clinicians; and asking clinicians to make rationing judgements at the point of care is liable to undermine patient trust.

## Abbreviations

EBM: Evidence-based medicine; ICER: Incremental cost-effectiveness ratio; NICE: National Institute for Health and Clinical Excellence; RSV: Respiratory syncytial virus.

## Competing interests

*Financial competing interests*: None

*Non-financial competing interests*: I worked from 1997 to 2008 as Director of the West Midlands Health Technology Assessment Collaboration, which produces systematic reviews and economic evaluations for health care policy makers, e.g. NICE, to inform policy decisions.

## Authors' contributions

AB is the sole author of this paper and the views expressed are hers and are not intended to represent the views of any organisation with which she is or has been associated.

## Pre-publication history

The pre-publication history for this paper can be accessed here:

http://www.biomedcentral.com/1472-6963/10/184/prepub
